# The Importance of Real-World Data in Evaluating the Safety of Biosimilars: A Descriptive Study of Clinical Practice in an Oncohematological Italian Population

**DOI:** 10.3390/cancers16193419

**Published:** 2024-10-08

**Authors:** Silvana A. M. Urru, Flavia Mayer, Stefania Spila Alegiani, Francesca Paoloni, Anna Guella, Roberta Murru, Giampaolo Bucaneve, Giulio Formoso, Vito Racanelli, Isacco Ferrarini, Claudio Fozza, Giuseppe Longo, Felice Musicco, Annalisa Campomori

**Affiliations:** 1 Hospital Pharmacy Unit, Trento General Hospital, Autonomous Province of Trento, 38122 Trento, Italy; annalisa.campomori@apss.tn.it; 2 Pharmacoepidemiology Unit, National Center for Drug Research and Evaluation, Italian National Institute of Health, 00161 Rome, Italy; flavia.mayer@iss.it (F.M.); stefania.spila@iss.it (S.S.A.); 3 Fondazione GIMEMA Onlus, 00187 Rome, Italy; f.paoloni@gimema.it; 4 Section of Hematology and Stem Cell Transplantation, Department of Medicine, APSS Trento, Autonomous Province of Trento, 38122 Trento, Italy; anna.guella@apss.tn.it; 5 Hematology and Stem Cell Transplantation Unit, Ospedale Oncologico A. Businco, ARNAS G. Brotzu, 09134 Cagliari, Italy; roberta.murru@aob.it; 6 Ospedale S. Maria della Misericordia, 06129 Perugia, Italy; gbucaneve@regione.umbria.it; 7 Azienda USL—IRCCS di Reggio Emilia, 42122 Reggio Emilia, RE, Italy; giulio.formoso@ausl.re.it; 8 Department of Medicine, APSS Trento, Autonomous Province of Trento, 38122 Trento, Italy; vito.racanelli@apss.tn.it; 9 Section of Haematology, Department of Medicine, University of Verona, 37134 Verona, Italy; isacco.ferrarini@univr.it; 10 Department of Clinical and Experimental Medicine, University of Sassari, 07100 Sassari, Italy; cfozza@uniss.it; 11 Oncological Medicine Unit, Azienda Ospedaliera Universitaria di Modena, 41125 Modena, Italy; longo@unimore.it; 12 Regina Elena San Gallicano IRCCS di Roma, 00144 Roma, Italy; felice.musicco@ifo.it

**Keywords:** biosimilars, rituximab, safety, oncohematology, non-Hodgkin lymphoma (NHL), chronic lymphocytic leukemia (CLL)

## Abstract

**Simple Summary:**

This study analyzes the use of rituximab (RTX) in the daily clinical practice of several Italian oncohematology centers, with a particular focus on the adoption of biosimilars. The results show that only a minority of patients (22%) switched between different biosimilars, while the majority continued treatment with the biosimilars Rixathon and Truxima. This finding suggests that the proactive sharing of guidelines between regulators and prescribers, from the outset, may be an effective strategy to further promote the adoption of biosimilars. This approach could be particularly useful in those areas where the use of biosimilars is still lower than the national average, helping to free up economic resources that could be redirected towards other healthcare opportunities.

**Abstract:**

The clinical safety and efficacy of rituximab biosimilars compared to the reference rituximab (Mabthera) have been well established in randomized trials. However, concerns persist regarding the safety of changing from the reference product to biosimilars, and particularly between different biosimilars. This prospective multicenter observational study was conducted in 13 oncohematology units of eight Italian regions. The study included 800 patients with non-Hodgkin lymphoma (NHL) or chronic lymphocytic leukemia (CLL) who received rituximab between March 2018 and June 2022. To minimize survivorship bias, only newly diagnosed patients (i.e., those without prior rituximab treatment) were included in the analysis of adverse drug reactions (ADRs). Thus, this study focused on 505 incident cases (79.8% of the initial cohort) from 13 centers. A total of 3681 rituximab infusions were administered, and 16.8% of the patients experienced at least one ADR. These were observed most frequently during the first infusion (44 patients, 52%) and the second infusion (17 patients, 20%). The most frequent reactions were general disorders and administration site conditions (n. 50, 8% serious). These findings support the clinical safety of rituximab biosimilars and suggest that switching between biosimilars does not increase the risk of adverse events. This evidence may alleviate concerns about biosimilar use, potentially leading to broader acceptance and reduced healthcare costs.

## 1. Introduction

Biologic medications, or biologics, are complex macromolecular drugs produced using living systems. The advent of targeted biologics has significantly transformed the treatment of various severe and chronic diseases [1]. The rapid development of these drugs has played a crucial role in advancing treatment strategies for conditions such as cancer (using monoclonal antibodies), autoimmune disorders, diabetes (with human insulin), and anemia (through erythropoietin substitutes) [2].

As defined by the EMA, a biosimilar is defined as a biologic medicinal product that is similar to another biologic medicine that has already been authorized for use.

The regulatory requirements for the approval of biosimilars are generally consistent across the guidelines issued by the EMA, WHO, and FDA [3,4,5]. All necessitate a stepwise approach to establish biosimilarity. These established regulatory pathways incorporate comparative assessments that involve analytical, non-clinical, and clinical studies. The regulations require head-to-head comparative studies for structural characterization, functional in vitro assays, pharmacokinetic and pharmacodynamic evaluations, and assessments of safety, efficacy, and immunogenicity. Biosimilarity is demonstrated based on the totality of the evidence across all evaluations, with each step being supported by the preceding one in the process.

The introduction of biosimilars into clinical practice has significantly reduced treatment costs and increased access to essential therapies. Rituximab biosimilars, in particular, have been widely adopted for treating hematologic malignancies.

Several randomized trials have confirmed that the reference product rituximab (Mabthera^®^) and its biosimilars exhibit similar clinical effectiveness and safety profiles.

However, despite their proven safety, many clinicians remain hesitant to switch patients from the reference product to a biosimilar due to concerns about immunogenicity or adverse reactions, even though studies show that biosimilars and reference products share the same efficacy.

For these reasons, and in light of the entry of the first rituximab biosimilar into the market, the Hospital Pharmacy and the Department of Hematology of Trento Hospital conducted a pilot observational study in 2018. The aim was to collect new safety information and to accompany clinicians and patients in this cultural transition. This pilot study [6] was selected and included by the Italian Medicines Agency (AIFA) within a pharmacovigilance project on the use of biological products and biosimilars in Italy (VALORE Project) [7]. Since January 2020, several other hematology units and hospital pharmacist units of different Italian regions have participated in the same observational study.

## 2. Objective

The purpose of this study was to collect clinical information about any adverse drug reaction (ADR) related to the use of rituximab (originator or biosimilars) and to the practice of switching among different products in patients affected by oncohematological diseases, particularly non-Hodgkin lymphoma (NHL), chronic lymphocytic leukemia (CLL), and indications included in the list provided by Law 648/96 [8] (Law 648/96 is one of the early-access schemes in Italy that allows the supply of specific drugs that are not yet available or undergoing clinical trials).

## 3. Methods

### 3.1. Study Population

This prospective multicenter observational study was conducted in 13 oncohematology units of eight Italian regions. The study population consisted of adult patients diagnosed with NHL and CLL who were consecutively admitted to the hematology departments of participating centers between 10 March 2018, and 10 June 2022, and whose treatment included the administration of rituximab originator, both intravenously (IV, Mabthera^®^ intravenous—MabIV) and subcutaneously (SC, Mabthera^®^ subcutaneous—MabSC), as well as IV rituximab biosimilars (specifically Truxima^®^ (Tru) or Rixathon^®^ (Rix)).

Treatment decisions were made at the clinician’s discretion, in accordance with the Summary of Product Characteristics (SPC) labeling information (rituximab administered at a dose of 375 mg/m^2^ body surface area, once every 3 weeks, or subcutaneously at a dose of 1400 mg) and standard clinical practice.

### 3.2. Data Collection

Baseline clinical data were obtained from patient records and included clinical information (e.g., age, sex, diagnosis, number of comorbidities, disease duration), concomitant medications, previous lines of treatment (chemotherapy, radiotherapy, or other pharmacological treatment), number of previous cycles of RTX, and quality of life.

The 42-item Functional Assessment of Cancer Treatment-Lymphoma (FACT-Lym) questionnaire was used to assess aspects of HRQoL (results will soon be published).

Routine clinical evaluations, including disease activity measurements, were conducted three months after the administration of rituximab biosimilars and approximately every 4–6 months thereafter.

Follow-up data were collected up to February 2023. For those who discontinued RTX, the reason for discontinuation [lack of efficacy (according to clinician judgment accompanied by disease activity assessment), ADRs, death, or other (new contraindication, no longer indicated)], date of discontinuation (start date of new therapy, date of death, or date of decision to stop RTX for other reason), and—where applicable—subsequent treatment were recorded.

All study data were collected and managed using REDCap [9] (Research Electronic Data Capture) tools hosted at the GIMEMA (Gruppo Italiano Malattie EMatologiche dell’Adulto) Foundation.

### 3.3. Clinical Outcomes

The outcomes, with respect to safety, were the proportion of patients experiencing at least one adverse reaction and the proportion of patients experiencing at least one grade 3 or higher ADR.

### 3.4. Statistical Analysis

During the study period, patients were classified according to the treatment received: (a) single product, i.e., either a biosimilar or an originator (no switcher group), or (b) two or more different products, i.e., different biosimilars, different originators, originators and biosimilars (switcher group).

Categorical variables were summarized as frequencies with corresponding percentages, while continuous variables were described using either means and standard deviations (if normally distributed) or medians and interquartile ranges (IQRs).

Differences were evaluated through Student’s t-test or the Kruskal–Wallis test for continuous variables and the chi-squared test for categorical variables. The p-value was set to 0.05.

Patterns of switching were represented through a Sankey diagram, which allowed us to display the proportion of patients who changed their treatment during the study period. All statistical analyses were performed using R (R core team 2021).

## 4. Results

**Figure 1:**
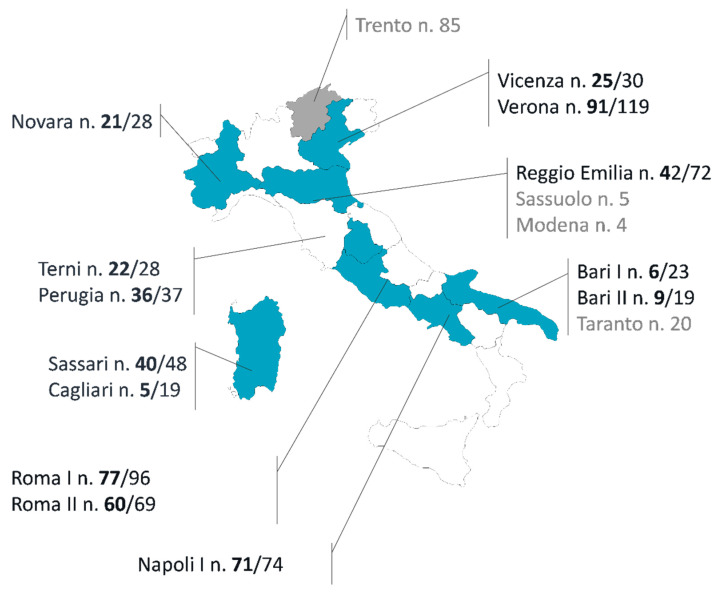
Blue: geographic distribution of participating hematology centers and patient enrollment in Italy (505 analyzed out of 800 enrolled). Black: participating hematology centers included in the study (number of naïve patients/number of enrolled patients); gray: participating hematology centers not included in the study (number of enrolled patients); n.: number.

**Figure 2:**
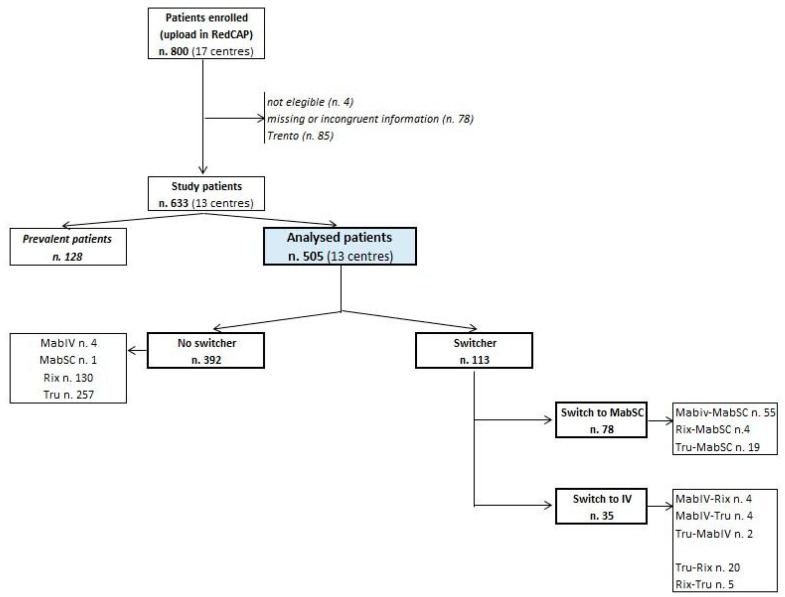
Flowchart of the study: n.: number; ADR: adverse drug reaction; MabIV: Mabthera^®^ intravenous; MabSC: Mabthera^®^ subcutaneous; Rix: Rixathon^®^; Tru: Truxima^®^; IV: intravenous.

A total of 800 participants were recruited from 17 hematology units of nine Italian regions (Figure 1). Data on 85 patients treated in Trento Hospital had already been published (pilot study) [6]; therefore, they were excluded from the present study. Three other centers (n = 82 patients) were also excluded because they had too few patients and incongruent and/or missing data. To minimize survivorship bias, only naïve patients (i.e., without prior administrations of RTX) were considered in the analysis of ADRs. Therefore, among the 633 patients included in the study, we focused our analysis on 505 (79.8%) incident cases from 13 centers (Figure 2).

The 505 patients (42% women) included in this study were affected by NHL (n = 453), CLL (n = 33), or one of the diseases included in the Italian Law 648/96 list (n = 19). The patients had a median age at baseline of 66.8 years (interquartile range (IQR): 57.5–73.9 years), and 41.6% had a performance status of 0. The median follow-up of the patients was 317 days (IQR 217–461 days) (Table 1).

During the study period, the patient population received 3681 infusions of rituximab: 53% (n = 1984) Tru, 24% (n = 901) Rix, 19% (n = 694) MabSC, and 3% (n = 102) MabIV. The mean number of infusions per patient was 7.3 (SD 3.8).

### 4.1. Rituximab Treatment

Of the 505 naïve patients, 392 (78%) did not experience any switch (“no switcher” group; n = 257 on Tru and n = 130 on Rix), and 113 (22%) were “switcher” patients (Table 1). Overall, 57% of patients were treated with a combination of RTX and chemotherapy (64% in “no switcher” patients and 32% in “switcher” patients); 160 (55.6%) of these had a diagnosis of aggressive NHL. Twenty-five patients were treated with RTX monotherapy (23 “no switcher” patients), among whom 88% were affected by NHL.

**Table 1:** Rituximab infusions and schedule in the 505 naïve patients by switch.

	All Patients	n. RTX Infusions		Schedule *		
		Mean ± sd	Median [IQR]	RTX + Chemotherapy	RTX Monotherapy	RTX + Chemotherapy/ RTX Monotherapy
No switch during the study period	392	6.4 ± 2.7	6.0 [6.0–8.0]	252 (64.3%)	23 (5.9%)	111 (28.3%)
Originator—Mabthera IV	4	7.3 ± 3.9	6.0 [5.0–9.5]	3 (75.0%)	0 (0.0%)	1 (25.0%)
Originator—Mabthera SC	1	7.0	7.0	1 (100.0%)	0 (0.0%)	0 (0.0%)
Biosimilar—Rixathon	130	5.8 ± 2.4	6.0 [4.0–7.0]	101 (77.7%)	3 (2.3%)	25 (19.2%)
Biosimilar—Truxima	257	6.7 ± 2.8	6.0 [6.0–8.0]	147 (57.2%)	20 (7.8%)	85 (33.1%)
Switch during the study period	113	10.5 ± 5.3	8.0 [6.0–15.0]	36 (31.9%)	2 (1.8%)	74 (65.5%)
sw protocol	57	10.4 ± 5.5	8.0 [6.0–15]	17 (29.8%)	0 (0.0%)	40 (70.2%)
sw or	2	13.0 ± 7.1	13.0 [8.0–18.0]	0 (0.0%)	0 (0.0%)	2 (100.0%)
sw ob	21	12.5 ± 5.4	13.0 [8.0–17.0]	4 (19.0%)	0 (0.0%)	17 (81.0%)
sw bb	25	8.9 ± 4.3	8.0 [6.0–10.0]	10 (40.0%)	2 (8.0%)	12 (48.0%)
sw bo	8	10 ± 5.4	8.5 [7.0–12.5]	5 (62.5%)	0 (0.0%)	3 (37.5%)
Total	505	7.3 ± 3.8	6.0 [6.0–8.0]	288 (57.0%)	25 (5.0%)	185 (36.6%)

* Seven cases without information on schedule; n.: number; sd: standard deviation; IQR: interquartile range; RTX: rituximab; sw protocol: switch to MabSC; sw or: switch among originators; sw ob: switch from originator to biosimilar; sw bb: switch among biosimilars; sw bo: switch from biosimilar to originator.

**Figure 3:**
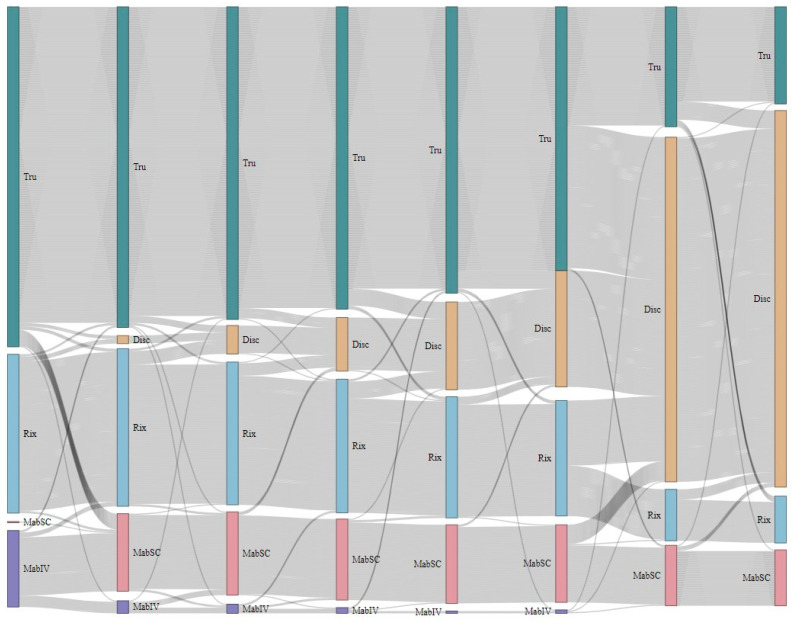
Sankey diagram of 505 naïve patients for the first eight infusions. For each patient, the description of the rituximab treatments at each infusion is graphically reported. MabIV: Mabthera^®^ intravenous; MabSC: Mabthera^®^ subcutaneous; Rix: Rixathon^®^; Tru: Truxima^®^; Disc: discontinuation.

In the first infusion, the majority of the 505 naïve patients started treatment with the biosimilars Tru (n. 298) and Rix (n. 139), while 67 patients started with the originator MabIV. The Sankey diagram (Figure 3) shows the RTX treatments of the 505 patients from the first to the eighth infusion, after which 40% of the patients were still in treatment. Notably, 69% of “switcher” patients (n = 78) switched to MabSC, with the majority (73%) switching at the second infusion after initially receiving MabIV (40 patients), Tru (15 patients), or Rix (2 patients) at the first infusion.

From the Sankey diagram (Figure 3), two types of switches can be highlighted: the first is the switch to MabSC, and the second is the switch among different IV RTX treatments. Comparing these two groups with the “no switcher” patients (Table 2), it becomes evident that the baseline characteristics (i.e., at the first infusion, which does not necessarily coincide with the infusion at which the switch occurs for “switcher” patients) are similar, except for the duration of the disease (for “no switcher” patients, the median is 1 year, for “switchers” to MabSC, it is 1.5 years, and for “switchers” between IV rituximab treatments, it is 2 years), the type of diagnosis (the majority of “switchers” to MabSC (63%) and “no switcher” patients (47%) had aggressive NHL, while nearly half (49%) of the “switchers” between IV rituximab treatments had unspecified NHL), the follow-up time (for “no switcher” patients, the median is nine and a half months, for “switchers” to MabSC, it is one year and three months, and for “switchers” between IV rituximab treatments, it is one and a half years), and the number of infusions (for “no switcher” patients, the average number is six, whereas “switcher” patients had an average of ten infusions).”

The characteristics of patients who discontinued RTX treatment before the six th cycle are shown in Table S1. For the majority of these patients (70%), the reason for interrupting treatment was not given, while 21.8% interrupted the treatment due to ADRs, and 7.9% for other reasons (mostly due to the progression of the disease).

### 4.2. Adverse Reactions Related to Rituximab

Overall, 85 (16.8%) patients reported ADRs to RTX. ADRs occurred more frequently in patients with indolent NHL (18.8%), compared to those with highly malignant NHL (15.6%), those with CLL (15.2%), and those with conditions treated under Law 648-1996 (15.8%). A total of 21 ADRs occurred in 113 patients (18.8%) with unspecified NHL (Table 3).

The correlations between ADRs and the demographic and clinical–laboratory characteristics of the 505 naïve patients are shown in Table S2. The occurrence of ADRs was significantly associated with the age at diagnosis (*p* = 0.05) and a higher neutrophil count (*p* < 0.01). In the 85 patients who experienced at least one ADR during the study period, 124 RTX-related events were reported (1.5 events/patient). Among these, 16% were classified as grades 3–5. The most frequent reactions were general disorders and administration site conditions (n. 50, 8% serious) (Table 4).

In the study population, 16.8% of the patients experienced at least one ADR. This percentage varied significantly among the different treatment groups, with a higher incidence among the “no switcher” patients (20.7%)—particularly among those receiving Rix (24.6%) and Tru (17.9%)—and a lower incidence (3.5%) among the “switcher” patients (Table 3).

**Figure 4:**
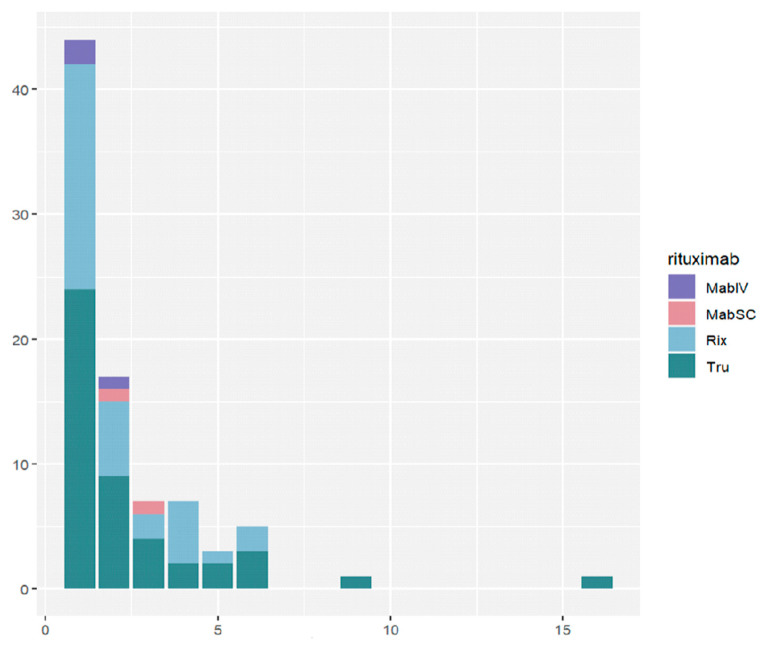
Number of patients with at least one ADR related to RTX, by number of infusions and type of RTX (n = 85). MabIV: Mabthera^®^ intravenous; MabSC: Mabthera^®^ subcutaneous; Rix: Rixathon^®^; Tru: Truxima^®^.

Among the 85 patients with at least one ADR to RTX in the 21 infusions, ADRs were observed most frequently during the first infusion (44 patients, 52%) and the second infusion (17 patients, 20%). Between the third and sixth infusions, fewer than 10 patients experienced ADRs, and from the seventh infusion onwards the number decreased to 1. Figure 4 shows that the majority of ADRs occurred in patients undergoing treatment with Rix or Tru.

The frequency of ADRs also diminished as the number of infusions increased. During the initial infusion, 9% of patients (44 out of 505) experienced ADRs, which dropped to 3.4% at the second infusion (17 out of 498 patients). From the third to the fifteenth infusion, the incidence remained below 2%. Notably, at the sixteenth infusion, there appeared to be a slight increase, but this was attributed to the limited number of patients receiving treatment at that stage, resulting in a less stable incidence estimate (3.1%, 1 patient out of 32). (Figure S1).

In Figure S2, we report the incidences of ADRs at each infusion separately for “switcher” and “no-switcher” patients. Of the 85 ADRs, 4 occurred in “switcher” patients, whereas 81 occurred in “no-switcher” patients. In “switcher” patients, the incidence did not reach 1% (n = 112), while in “no-switcher” patients the incidence at the first infusion was 11.2% (n = 393), dropped to 4.1% at the second infusion (n = 386), remained below 2% until the eighth infusion (n = 103), and appeared to increase again in the ninth and sixteenth infusions. However, as with the overall incidence, these fluctuations were probably due to the small number of patients receiving treatment from the ninth infusion onwards (n < 43).

## 5. Discussion

The protein structure of biologics and biosimilars makes them susceptible to molecular changes within the body through various biological pathways. As a result, certain safety concerns may only become apparent beyond the controlled clinical trial periods. This highlights the importance of post-approval safety monitoring and risk management for these drugs.

From this perspective, the proactive sharing of guidelines between regulatory bodies and prescribers plays a critical role in encouraging the adoption of biosimilars.

Indeed, while the clinical equivalence of rituximab biosimilars and the originator has been well established, concerns about the safety of switching persist. The present study, conducted across multiple Italian centers, shows that switching between biosimilars or from the originator to biosimilars did not lead to an increase in adverse drug reactions (ADRs). These findings are consistent with other studies that report no significant safety concerns following biosimilar switches. The clear and concise guidance shared between physicians and pharmacists early on likely also addresses potential concerns about safety, efficacy, and regulatory expectations, fostering greater confidence among prescribers.

However, this study underscores the need for ongoing pharmacovigilance, particularly in real-world settings, to ensure long-term safety as switching practices become more prevalent in clinical settings.

In contrast to clinical trials, this real-world evidence offers a more comprehensive understanding of how rituximab biosimilars perform in routine practice. Although the study demonstrates a favorable safety profile, it also reveals that switching is more commonly associated with subcutaneous formulations and that patients with aggressive non-Hodgkin lymphoma were the most likely to undergo such switches. These patterns reflect clinicians’ preference for the ease of administration of subcutaneous therapies in certain aggressive forms of lymphoma. Future studies could focus on the long-term impacts of switching on efficacy, as this study was primarily designed to evaluate safety.

This observational study, conducted in real-life settings across multiple Italian regions, demonstrates that switching between rituximab originator and biosimilars (Tru, Rix) and switching between different biosimilars are common practices in the treatment of oncohematological diseases. The findings from this study are consistent with previous research that confirms the safety of rituximab biosimilars and supports their use as an effective treatment option.

Switching from reference biologics to a biosimilar product, or between two biosimilars of the same reference biologic, is generally driven by affordability, formulary requirements, or the relocation of the patient. Usually, the use of a compound with similar safety and efficacy profile is less considered.

Biosimilars are therapeutic biological products that closely resemble the approved reference drug in terms of quality, safety, and efficacy. The reference drug typically corresponds to the original brand of drug [3,4]. It is crucial to understand that, unlike small-molecule chemicals, biologics cannot achieve complete “consistency” due to their intricate structure and manufacturing process. Even when produced by the same manufacturer, biologics may exhibit variations among different sources and distinct batches, or even within the same batch of products.

Even if these differences do not have a clinical impact, clinicians may be reluctant to use biosimilars or to switch a patient from the reference drug to its biosimilars.

Indeed, some open-label studies have shown an increased number of withdrawals or ADRs following a switch; these outcomes were less frequently observed in randomized studies, suggesting the potential occurrence of a “nocebo” effect resulting from negative expectations toward the biosimilar.

When it comes to switching to biosimilars, safety is always a top concern for clinicians, despite several studies having demonstrated that switching to a biosimilar is safe and effective. Moreover, many patients have reported improved outcomes and cost savings [10].

To reassure healthcare professionals and the public that the risk of immunogenicity-related safety concerns or diminished efficacy is unchanged after switching from a reference biologic to a biosimilar medicine, several studies have been conducted over the past decade [11].

Since our first real-life cohort study assessing the safety of switching between different rituximab formulations (biosimilars and originator) in NHL and CLL patients, two other studies have been conducted [12,13], including an RCT [14]. All of these studies demonstrate a closely related overlap in the efficacy, safety, and immunogenic profile of both drugs, consistent with the literature data on rituximab’s safety.

In spite of the fact that some open-label studies have shown an increase in the number of withdrawals or adverse events (AEs) after a switch, these events were less commonly noted in randomized studies [15,16], suggesting the possible manifestation of a “nocebo” effect due to negative expectations towards the biosimilar [17].

The strength of this study, conducted among 13 oncohematology units, undoubtedly lies in its providing a description of the use of rituximab in clinical practice in Italy, with a focus on the frequency of switches and adverse reactions. However, it is challenging to establish a correlation between the two, given that the majority of events occur after the first or second infusion.

Nevertheless, it is reassuring that those who switch do not experience more adverse reactions compared to those treated with the original formulation. Most switches are to the subcutaneous formulation, and the majority of these patients have an aggressive form of non-Hodgkin lymphoma.

The number of patients experiencing at least one ADR during the study period was less than 20%, and the most frequent events were general disorders and administration-site-related conditions, in accordance with the literature and the summary of product characteristics.

### Limits of This Study

The COVID-19 pandemic has had a serious and disruptive effect on the conduct of clinical trials in hematology and oncology, with both immediate and delayed consequences. In our prospective cohort study, which started at the very beginning of the COVID-19 pandemic, many patients were asked to delay their access to the clinic and less time was dedicated by health professionals to data collection and research compared to health assistance and clinical practice.

Moreover, the COVID-19 pandemic likely impacted treatment decisions, particularly in the later stages of the study. The pandemic introduced unprecedented challenges, including shifts in healthcare priorities, disruptions to supply chains, and the need for remote patient management, which may have affected both the availability of certain drugs and the physicians’ choices.

While our results align with trends observed in similar studies, particularly in Europe, the geographic focus on Italy may limit the broader applicability of our conclusions. For instance, regional variations in healthcare infrastructure, regulatory environments, and patient demographics could influence the outcomes. This localized focus may affect the generalizability of our results to other countries with different healthcare systems or population characteristics. Future studies should consider a broader geographic scope to validate these findings and explore the impact of regional differences more comprehensively.

## 6. Conclusions

After a median follow-up of 310 days, the adverse reactions reported were similar in terms of their seriousness and frequency, regardless of the rituximab formulation or switching.

The findings of this study reaffirm that the use of rituximab biosimilars, as well as switching between the originator and biosimilars, is safe and effective in real-life clinical practice for patients with oncohematological diseases.

Information gathered in real-world observational studies conducted during clinical practice can offer significant and valuable evidence that complements the findings of randomized controlled trials (RCTs) regarding the effectiveness and safety of biosimilars in various medical conditions and treatment scenarios. This is particularly important when switching patients from originators to considerably less expensive biosimilars, as well as when there are concerns regarding their effectiveness in practice.

## Figures and Tables

**Table 2:** Baseline characteristics of 505 naïve patients by switch.

Characteristics	All Patients	Switch to MabSC	Switch to IV (OR/BIO)	No Switchers	*p*
Number of patients	505	78	35	392	
Female [n. (%)]	212 (42.0%)	29 (37.2%)	12 (34.3%)	171 (43.6%)	0.36
BMI (kg/m^2^) [median (IQR)]	25.0 (22.5–27.6)	25.8 (22.8–28.3)	24.4 (21.9–28.1)	25.0 (22.5–27.3)	0.43
Age at diagnosis (years) [median (IQR)]	65.8 (57.0–73.3)	67.1 (56.1–73.4)	68.8 (59.4–73.8)	65.3 (57.1–73.2)	0.50
Age at baseline (years) [median (IQR)]	66.8 (57.5–73.9)	67.6 (57.1–73.8)	69.2 (59.8–74.1)	66.6 (57.4–74.0)	0.59
Duration of disease (days) [median (IQR)]	390.0 (276.0–637.0)	534.0 (388.0–787.0)	732.0 (358.0–965.5)	361.0 (265.0–544.0)	<0.001
Diagnosis					<0.001
Indolent non-Hodgkin lymphoma	96 (19.0%)	28 (35.9%)	5 (14.3%)	63 (16.1%)	
Aggressive non-Hodgkin lymphoma	244 (48.3%)	49 (62.8%)	10 (28.6%)	185 (47.2%)	
Unspecified non-Hodgkin lymphoma	113 (22.4%)	1 (1.3%)	17 (48.6%)	95 (24.2%)	
Chronic lymphocytic leukemia	33 (6.5%)	0 (0.0%)	2 (5.7%)	31 (7.9%)	
Law 648-96	19 (3.8%)	0 (0.0%)	1 (2.9%)	18 (4.6%)	
Time of follow-up (days) [median (IQR)]	317 (217–461)	445 (335–812)	498 (255–823)	295 (210–404)	<0.001
Number of concomitant medications					0.96
0	114 (22.6%)	20 (25.6%)	7 (20.0%)	87 (22.2%)	
1–3	246 (48.7%)	37 (47.4%)	17 (48.6%)	192 (49.0%)	
≥4	145 (28.7%)	21 (26.9%)	11 (31.4%)	113 (28.8%)	
Number of comorbidities					0.55
0	161 (31.9%)	25 (32.1%)	10 (28.6%)	126 (32.1%)	
1–2	179 (35.4%)	27 (34.6%)	17 (48.6%)	135 (34.4%)	
≥3	165 (32.7%)	26 (33.3%)	8 (22.9%)	131 (33.4%)	
Performance status [n (%)]					0.035
0	210 (41.6%)	39 (50.0%)	6 (17.1%)	165 (42.1%)	
1	156 (30.9%)	23 (29.5%)	15 (42.9%)	118 (30.1%)	
2	25 (5.0%)	1 (1.3%)	2 (5.7%)	22 (5.6%)	
≥3	14 (2.8%)	0 (0.0%)	1 (2.9%)	13 (3.3%)	
NA	100 (19.8%)	15 (19.2%)	11 (31.4%)	74 (18.9%)	
Number of infusions [mean]	7.3 ± 3.8	10.8 ± 5.3	9.7 ± 5.3	6.4 ± 2.7	<0.001

Notes: n.: number; IQR: interquartile range; RTX: rituximab; SC: subcutaneous; IV: intravenous; NA: not applicable.

**Table 3:** Occurrence frequencies of at least one adverse drug reaction by diagnosis and switch.

	All Patients	n. At Least 1 ADR	% At Least 1 ADR
Diagnosis			
Aggressive non-Hodgkin lymphoma	244	38	15.6
Unspecified non-Hodgkin lymphoma	113	21	18.6
Indolent non-Hodgkin lymphoma	96	18	18.8
Chronic lymphocytic leukemia	33	5	15.2
Law 648-96	19	3	15.8
No switch during the study period	392	81	20.7
Originator—Mabthera IV	4	3	75.0
Originator—Mabthera SC	1	0	0.0
Biosimilar–Rixathon	130	32	24.6
Biosimilar—Truxima	257	46	17.9
Switch during the study period	113	4	3.5
sw protocol	57	1	1.8
sw or	2	0	0.0
sw ob	21	1	4.8
sw bb	25	2	8.0
sw bo	8	0	0.0
Total	505	85	16.8

Notes: n.: number; ADR: adverse drug reaction; sw protocol: switch to MabSC; sw or: switch among originators; sw ob: switch from originator to biosimilar; sw bb: switch among biosimilars; sw bo: switch from biosimilar to originator.

**Table 4:** Adverse drug reactions related to rituximab.

Type of Events (Cases, n = 85)	ADRs Related to Rituximab				
	n. Any Grade	n. Grade 1–2	n. Grade 3–5	% Grade 1–2	% Grade 3–5
General disorders and administration site conditions	50	46	4	92.0	8.0
Respiratory, thoracic, and mediastinal disorders	16	15	1	93.8	6.3
Gastrointestinal disorders	12	10	2	83.3	16.7
Infections and infestations	11	9	2	81.8	18.2
Blood and lymphatic system disorders	8	2	6	25.0	75.0
Skin and subcutaneous tissue disorders	8	8	0	100.0	0.0
Nervous system disorders	6	4	2	66.7	33.3
Musculoskeletal and connective tissue disorders	5	4	1	80.0	20.0
Cardiac disorders	4	3	1	75.0	25.0
Eye disorders	1	1	0	100.0	0.0
Psychiatric disorders	1	1	0	100.0	0.0
Secondary cancer	1	0	1	0.0	100.0
Vascular disorders	1	1	0	100.0	0.0
Total	124	104	20	83.9	16.1

Notes: n.: number; ADR: adverse drug reaction.

## Data Availability

The data that support the findings of this study are available upon request from the corresponding author [S.U.]. The data are not publicly available because they contain information that could compromise the research participants’ privacy/consent.

## Supplementary Materials

The following supporting information can be downloaded at: https://www.mdpi.com/article/10.3390/cancers16193419/s1, Figure S1: Incidence of 85 ADRs related to RTX by number of infusions (n. 505). ADR: Adverse Drug Reaction; Figure S2: Incidence of 85 ADRs related to RTX by number of infusions and switch (n. 505). Panel A: no switch patients; panel B: switch patients. ADR: Adverse Drug Reaction. Table S1: Characteristics of 505 naïve patients by ADR related to RTX; Table S2: Characteristics of 505 naïve patients by number of cycles.
