# Supplementary material for: The Importance of Real-World Data in Evaluating the Safety of Biosimilars: A Descriptive Study of Clinical Practice in an Oncohematological Italian Population
Source: Cancers (Basel). 2024 Oct 8;16(19):3419. doi: 10.3390/cancers16193419 (PMC11476336; doi:10.3390/cancers16193419)
Supplement: Supplementary file 1 [file cancers-16-03419-s001.zip › cancers-3242824-supplementary.pdf]

Supplementary Material

Table S1. Characteristics of 505 naïve patients by ADR related to RTX.

		ADRs		
**Characteristics**	**All patients**	**No**	**Yes**	**p**
**Number of patients**	**505**	**420**	**85**	
**Female** [n (%)]	**212 (42.0%)**	179 (42.6%)	33 (38.8%)	0.52
**BMI** (kg/m^2) [median (IQR)]	**25.0 (22.5-27.6)**	25.1 (22.5-27.7)	24.8 (22.3-26.8)	0.32
**Age at diagnosis** (years) [median (IQR)]	**65.8 (57.0-73.3)**	66.0 (57.7-73.6)	65.0 (53.6-71.2)	0.046
**Age at baseline** (years) [median (IQR)]	**66.8 (57.5-73.9)**	66.9 (58.3-74.3)	66.4 (53.9-72.8)	0.085
**Duration of disease** (days) [median (IQR)]	**390.0 (276.0-637.0)**	398.0 (277.0-648.0)	354.0 (271.0-574.0)	0.49
**Diagnosis**				0.93
Indolent Non-Hodgkin Lymphoma	**96 (19.0%)**	78 (18.6%)	18 (21.2%)	
Aggressive Non-Hodgkin Lymphoma	**244 (48.3%)**	206 (49.0%)	38 (44.7%)	
Not specified Non-Hodgkin Lymphoma	**113 (22.4%)**	92 (21.9%)	21 (24.7%)	
Chronic lymphocytic leukemia	**33 (6.5%)**	28 (6.7%)	5 (5.9%)	
Law 648-96	**19 (3.8%)**	16 (3.8%)	3 (3.5%)	
**Time of follow-up** (days) [median (IQR)]	**310.5 (217.0-452.0)**	316.0 (221.0-460.0)	262.0 (198.0-418.0)	0.11
**Number of concomitant medications**				0.65
0	**114 (22.6%)**	93 (22.1%)	21 (24.7%)	
1-3	**246 (48.7%)**	203 (48.3%)	43 (50.6%)	
≥4	**145 (28.7%)**	124 (29.5%)	21 (24.7%)	
**Number of comorbidities**				0.97
0	**161 (31.9%)**	133 (31.7%)	28 (32.9%)	
1-2	**179 (35.4%)**	149 (35.5%)	30 (35.3%)	
≥3	**165 (32.7%)**	138 (32.9%)	27 (31.8%)	
**Performance status** [n (%)]				0.78
0	**210 (41.6%)**	173 (41.2%)	37 (43.5%)	
1	**156 (30.9%)**	134 (31.9%)	22 (25.9%)	
2	**25 (5.0%)**	21 (5.0%)	4 (4.7%)	
≥3	**14 (2.8%)**	11 (2.6%)	3 (3.5%)	
NA	**100 (19.8%)**	81 (19.3%)	19 (22.4%)	
**n. infusion** [mean⊚sd]	**7.3±3.8**	7.5±4.0	6.3±2.6	0.009
**Neutrophils** (n. 39) [median (IQR)]	**3.8 (2.6-5.5)**	3.9 (2.7-5.6)	3.2 (2.3-4.5)	0.009
**Hemoglobin** (n. 30) [median (IQR)]	**12.5 (11.0-13.7)**	12.5 (11.0-13.7)	12.8 (11.0-13.9)	0.47
**Platelets** (n. 30) [median (IQR)]	**212.0 (155.0-275.0)**	214.0 (161.0-275.0)	198.5 (138.0-271.0)	0.21
**White blood cell** (n. 30) [median (IQR)]	**6.2 (4.7-8.9)**	6.3 (4.8-8.9)	5.8 (4.5-8.7)	0.73
**Lymphocytes** (n. 66) [median (IQR)]	**1.4 (0.9-2.5)**	1.4 (0.9-2.3)	1.7 (1.0-3.1)	0.18
**Creatinine** (n. 56) [median (IQR)]	**0.8 (0.7-1.0)**	0.8 (0.7-1.0)	0.8 (0.7-1.0)	0.75

ADR: Adverse Drug Reaction; IQR: interquartile range; sd: standard deviation.

Table S2. Characteristics of 505 naïve patients by number of cycles.

Characteristics	All patients	n. Cycles≥6	n. Cycles<5	p
**Number of patients**	**505**	**404**	**101**	
**Female** [n (%)]	**212 (42.0%)**	177 (43.8%)	35 (34.7%)	0.095
**BMI** (kg/m^2) [median (IQR)]	**25.0 (22.5-27.6)**	25.0 (22.7-27.4)	25.3 (22.2-27.7)	0.95
**Age at diagnosis** (years) [median (IQR)]	**65.8 (57.0-73.3)**	66.0 (57.4-73.0)	65.0 (55.4-74.3)	0.71
**Age at baseline** (years) [median (IQR)]	**66.8 (57.5-73.9)**	66.7 (57.7-73.6)	68.0 (55.6-74.4)	0.69
**Duration of disease** (days) [median (IQR)]	**390.0 (276.0-637.0)**	398.0 (289.0-624.0)	361.0 (202.0-675.0)	0.064
**Diagnosis**				0.005
Indolent Non-Hodgkin Lymphoma	**96 (19.0%)**	85 (21.0%)	11 (10.9%)	
Aggressive Non-Hodgkin Lymphoma	**244 (48.3%)**	192 (47.5%)	52 (51.5%)	
Not specified Non-Hodgkin Lymphoma	**113 (22.4%)**	94 (23.3%)	19 (18.8%)	
Chronic lymphocytic leukemia	**33 (6.5%)**	20 (5.0%)	13 (12.9%)	
Law 648-96	**19 (3.8%)**	13 (3.2%)	6 (5.9%)	
**Time of follow-up** (days) [median (IQR)]	**310.5 (217.0-452.0)**	317.0 (224.0-462.0)	255.0 (164.0-401.0)	0.003
**Number of concomitant medications**				0.48
0	**114 (22.6%)**	90 (22.3%)	24 (23.8%)	
1-3	**246 (48.7%)**	202 (50.0%)	44 (43.6%)	
≥4	**145 (28.7%)**	112 (27.7%)	33 (32.7%)	
**Number of comorbidities**				0.074
0	**161 (31.9%)**	124 (30.7%)	37 (36.6%)	
1-2	**179 (35.4%)**	153 (37.9%)	26 (25.7%)	
≥3	**165 (32.7%)**	127 (31.4%)	38 (37.6%)	
**Performance status** [n (%)]				0.35
0	**210 (41.6%)**	174 (43.1%)	36 (35.6%)	
1	**156 (30.9%)**	125 (30.9%)	31 (30.7%)	
2	**25 (5.0%)**	21 (5.2%)	4 (4.0%)	
≥3	**14 (2.8%)**	9 (2.2%)	5 (5.0%)	
NA	**100 (19.8%)**	75 (18.6%)	25 (24.8%)	
**Schedule** [n (%)]				<0.001
RTX+Chemotherapy	**288 (57.0%)**	204 (50.5%)	84 (83.2%)	
RTX monotherapy	**25 (5.0%)**	15 (3.7%)	10 (9.9%)	
RTX+Chemotherapy/monotherapy	**185 (36.6%)**	178 (44.1%)	7 (6.9%)	
NA	**7 (1.4%)**	7 (1.7%)	0 (0.0%)	
**at least 1 AE**				0.14
No	**420 (83.2%)**	341 (84.4%)	79 (78.2%)	
Yes	**85 (16.8%)**	63 (15.6%)	22 (21.8%)	
**Switch** [n (%)]				0.009
No switchers	**392 (77.6%)**	303 (75.0%)	89 (88.1%)	
Switch to iv (OR/BIO)	**35 (6.9%)**	29 (7.2%)	6 (5.9%)	
Switch to Mabsc	**78 (15.4%)**	72 (17.8%)	6 (5.9%)	
**RTX treatment** [n (%)]				0.004
originator-Mabthera IV (MabIV)	**4 (0.8%)**	3 (0.7%)	1 (1.0%)	
originator-Mabthera SC (MabSC)	**1 (0.2%)**	1 (0.2%)	0 (0.0%)	
biosimilar-Rixathon (Rix)	**130 (25.7%)**	87 (21.5%)	43 (42.6%)	
biosimilar-Truxima (Tru)	**257 (50.9%)**	212 (52.5%)	45 (44.6%)	
sw protocol	**25 (5.0%)**	23 (5.7%)	2 (2.0%)	
sw or	**8 (1.6%)**	7 (1.7%)	1 (1.0%)	
sw ob	**21 (4.2%)**	17 (4.2%)	4 (4.0%)	
sw bb	**2 (0.4%)**	2 (0.5%)	0 (0.0%)	
sw bo	**57 (11.3%)**	52 (12.9%)	5 (5.0%)	

n.: number; IQR: interquartile range; IV: intravenous; SC: subcutaneous; sw protocol: switch to Mabsc; sw or: switch among originators; sw ob: switch from originator to biosimilar; sw bb: switch among biosimilars; sw bo: switch from biosimilar to originator.

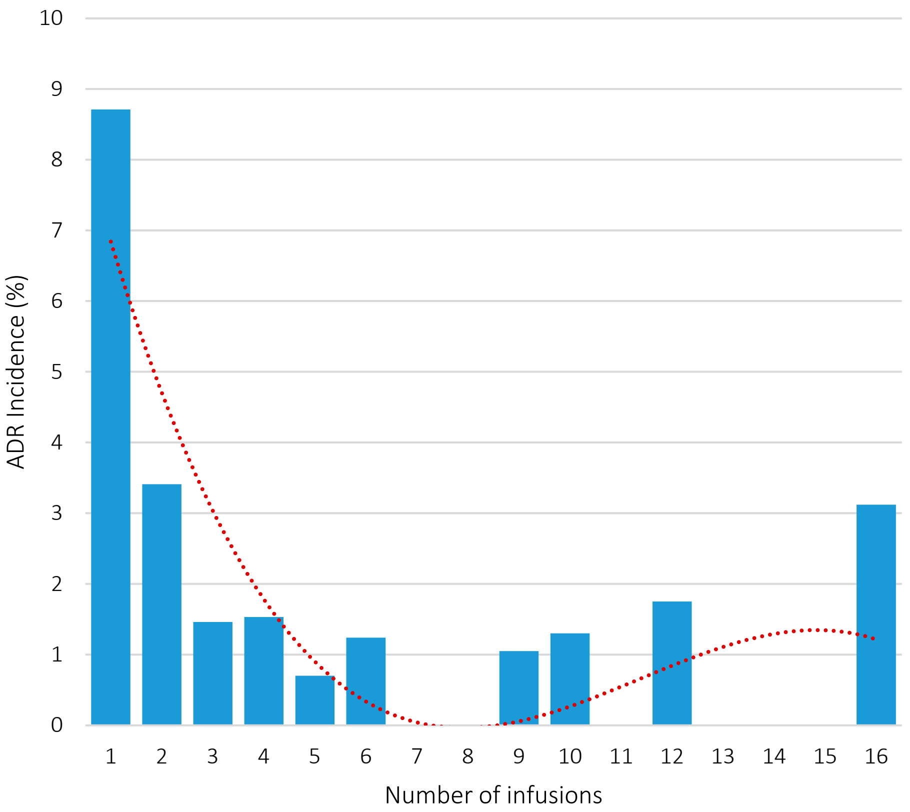

Figure S1. Incidence of 85 ADRs related to RTX by number of infusions (n. 505). *ADR: Adverse Drug Reaction.*

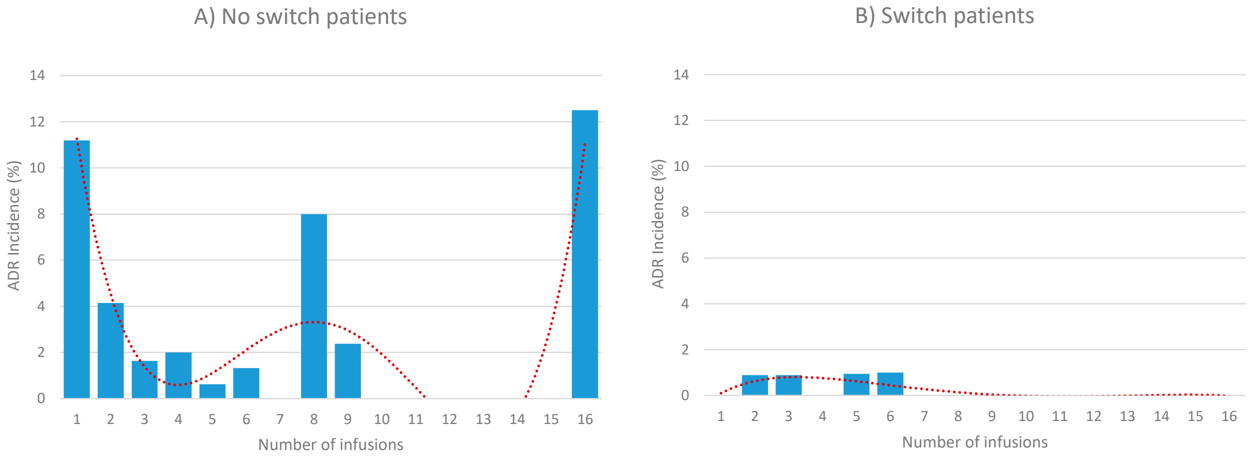

Figure S2. Incidence of 85 ADRs related to RTX by number of infusions and switch (n. 505). Panel A: no switch patients; panel B: switch patients. *ADR: Adverse Drug Reaction.*
